# Dynamic representations of theory testing in physical activity using ecological momentary assessment: an example guide utilizing multi-process action control

**DOI:** 10.3389/fpsyg.2025.1547090

**Published:** 2025-03-28

**Authors:** Ryan E. Rhodes, Matthew Y. W. Kwan

**Affiliations:** ^1^School of Exercise Science, Physical and Health Education, University of Victoria, Victoria, BC, Canada; ^2^Child and Youth Studies, Brock University, St. Catharines, ON, Canada

**Keywords:** habit, identity, behavioral regulation, hedonic motivation, opportunity, exercise, intention-behavior gap

## Abstract

Behavioral theories are essential in understanding physical activity (PA) and developing effective intervention strategies, yet most theories have been developed alongside common research methods available at their inception. Contemporary data collection methods such as intensive longitudinal designs (e.g., Ecological Momentary Assessment; EMA) are beginning to facilitate more advanced approaches to theorizing. One of the primary challenges in applying traditional behavior change theories, however, relates to measurement, as traditional multi-item measures are not practical nor may they accurately capture the dynamic elements of the construct sought in intensive longitudinal sampling. The purpose of this paper was to provide a user's guide of measures of the Multi-Process Action Control (M-PAC) Framework for use in EMA, followed by preliminary working examples. EMA offers opportunities to sample and obtain real-time (or near real-time) information that include processes that are more automatically or immediately activated in response to environmental stimuli or informational cues. As a result, we propose a slight re-operationalization of M-PAC as it relates to the interacting psychological systems in determining PA. We outline some of the measurement challenges with M-PAC using EMA, and the opportunities to blend more traditional and contemporary real-time approaches to advance theory and our understanding of PA. Together, this paper is intended to be a starting point, acknowledging the need to adapt traditional behavioral theories to incorporate the dynamic factors in determining PA. By doing so, we can advance our understanding of PA and develop more effective, and theory-based, interventions tailored to individual needs and contexts.

## Introduction

Physical activity (PA) is an essential behavior for health promotion (World Health Organization, 2022), yet a high prevalence of inactivity (Strain et al., 2024) underscores the necessity for interventions to promote sustained engagement. Behavioral theories are essential in understanding PA and developing effective intervention strategies (Rothman, 2004; Rhodes and Nigg, 2011; Michie et al., 2014). Theories offer a framework for outlining factors influencing PA and guiding application of underlying behavior change techniques for intervention (Teixeira et al., 2020; Connell et al., 2019; Carey et al., 2019; McEwan et al., 2019).

Most traditional behavioral theories (see Rebar and Rhodes, 2020), however, were developed alongside the common research methods available at their inception. These methods have often included cross-sectional, limited longitudinal, or experimental designs where prediction and evaluation of both behavior and its theoretical antecedents are relatively static with infrequent assessments (Dunton, 2017). When an understanding of gradual patterns of change between-people is sought, this methodological approach is likely to yield the desired outcome; however, it provides limited information on within-person changes or patterns of dynamic change and fluctuation (Conroy et al., 2020).

Contemporary data collection methods and intensive longitudinal designs such as Ecological Momentary Assessment (EMA) have enabled more advanced approaches to theorizing (Conroy et al., 2020) and analysis (Ruissen et al., 2021). These approaches help to move beyond nomothetic assumptions of behavioral theories at the population level to the idiographic nature of behavioral dynamics at the individual level. Specifically, EMA involves real-time (or near real-time) sampling of behaviors and experiences in natural environments, allowing researchers to capture time- and spatially-varying factors associated with PA (Stone and Shiffman, 1994). The emerging literature has begun to establish variability within theoretical constructs and behaviors across different temporal frames (Ruissen et al., 2022; Maher et al., 2017; Dunton, 2018).

One of the primary challenges in applying traditional behavior change theories to dynamic models, however, is measurement (Dunton, 2017). Traditional measures often require participants to reflect on the aggregate of their experiences and evaluations across a defined (and often long) period of time. Moreover, these measures were designed to include multi-items to improve the reliability of the assessment (Tabachnick and Fidell, 2012), and are akin and aligned with the nomothetic aims of traditional theories and the relatively static and infrequent assessment schedules. They pose a significant challenge when being applied to intensive longitudinal designs. First, from a practical perspective, EMA is a participant burden-heavy method, which by design requires a person to complete a series of questionnaires within a frequent sampling schedule (i.e., daily or multiple times within each day)—often precluding the inclusion of full-scale multi-item measures. It is simply not feasible to ask participants to answer long questionnaires with the high frequency of EMAs administered (Dunton, 2017; Wang et al., 2025). Second, and more nuanced, traditional measures that ask participants to reflect on a phenomenon may not accurately capture the dynamic element or momentary nature of the construct sought in intensive longitudinal sampling. For instance, constructs that have been developed as stable predictors of behavior in traditional theories may not lend to any advances with EMA studies because their properties are not theorized to change under moment-to-moment conditions. This may be one reason why EMA studies have tended to focus on affect and environmental-contextual factors in understanding health behaviors (Hartson et al., 2023). To effectively apply behavioral theories to dynamic models, it will require adaption of what may be a nomothetic construct to a straightforward single-item idiographic measure capturing the dynamical aspect of the theoretical construct in question.

The purpose of this paper is to provide an overview and initial user's guide of measures based on the Multi-Process Action Control (M-PAC) framework (Rhodes, 2021, 2017) for use in EMA, including working examples with prior data for its application. Like many theoretical approaches, M-PAC was conceptually developed with traditional nomothetic considerations to assessment and analyses; thus, reconfiguration of its measurement that is consistent with a more ideographic and dynamic operationalization is needed for guidance when considering EMA and intensive longitudinal analyses.

## An overview of M-PAC

M-PAC was designed as a meta-construction of PA behavior change from an initial decision to sustained behavioral patterns (see Rhodes, 2017, 2021). The majority of its application involves nomothetic cross-sectional or longitudinal evaluations across long periods of time (Rhodes, 2024). Recommended measures within M-PAC reflect traditional research approaches, assessing the aggregate of experiences. But given the proliferation of smartphones and software enabling intensive longitudinal designs, and real-time assessments of individual thoughts and momentary reflections, adaptations to theoretical frameworks such as M-PAC are needed.

Overall, M-PAC involves three connected, layered, yet progressive psychological processes that subsequently co-determine a sustained PA pattern. These processes can be modified by specific external behavior change techniques, but naturally build upon and co-determine each other through new and repeated experiences (Rhodes, 2021)—thus making a dynamical model appropriate to explore such variation. *Reflective processes* in M-PAC (affective judgments, instrumental attitude, perceived opportunity, and perceived capability) represent the consciously deliberated expectations of performing PA, that culminate in a decisional intention to engage in behavior. Enacting initial intention, however, is marked by *regulatory processes*, which represent behavioral, cognitive, and affective regulation tactics. Finally, *reflexive processes* in M-PAC are constructs that develop as a consequence of repeated intention-PA coupling across time and drive sustained behavior (Rhodes, 2017). M-PAC includes habit (learned cue-behavior associations) and identity (role self-categorization) as key reflexive constructs (Rhodes et al., 2021; Rhodes, 2021).

## Adapting M-PAC for dynamic modeling and EMA studies

**Figure 1:**
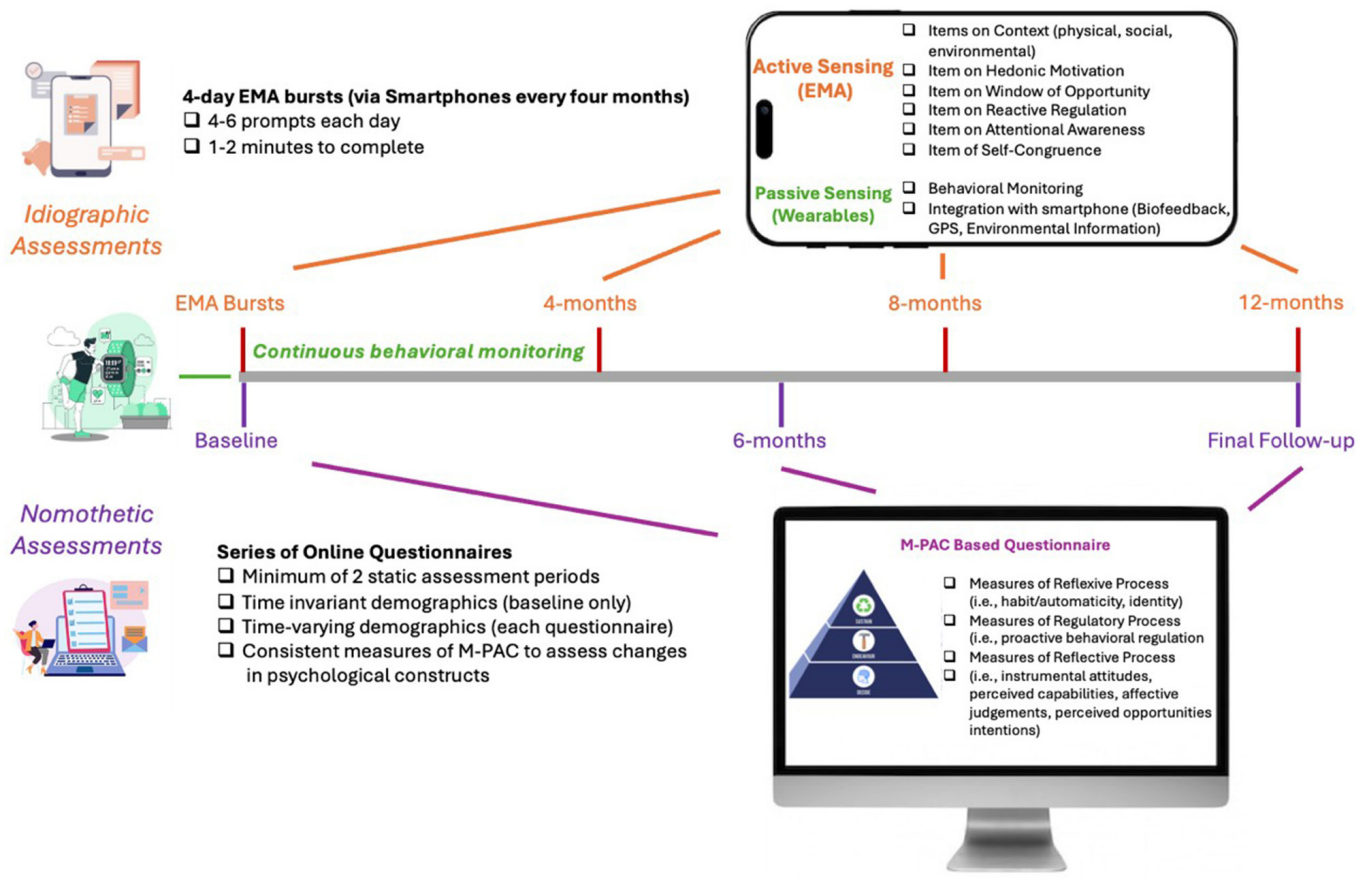
Example research design and checklist to applying idiographic and nomothetic assessments to the multi-process action control framework. This represents one example of a one-year intensive longitudinal design that incorporates both dynamic assessments of M-PAC constructs (through four 4-day EMA bursts) alongside more stable anticipatory and retrospective assessments of the reflective, regulatory and reflexive processes (through baseline, and 6- and 12-month follow-ups).

M-PAC holds conceptual promise for EMA research due to its layered and blended representation of processes determining behavior, including a temporal element moderating the strength of each relationship over time. The challenge, however, lies in the re-operationalization of its constructs that are both practical and feasible for EMA research. In the following sections, we address each M-PAC construct, discussing its conceptual origins and recommendations to better reflect the dynamic within-person components applicable for EMA research (see Table 1; Supplementary Table 1). We then include a proposed schematic integrating traditional and dynamic assessments for theory-testing using M-PAC (Figure 1).

**Table 1:** Re-operationalized M-PAC framework and recommended assessments.

**M-PAC construct**	**Generalized representation**	**Dynamic representation**	**Rationale**	**Recommendations for EMA**
**Reflective process**				
Instrumental attitude	Evaluation of perceived usefulness or practicality of a behavior	Not intended to be dynamic	Construct is meant to be a consideration of repeated behavior over longer duration to assess perceived benefits	Not recommended for EMA
Affective judgments	Evaluation of the perceived pleasure of a behavior or behavioral experience	Not originally intended to be dynamic but see hedonic motivation (below)	Construct is meant to be a consideration of repeated behavior over longer duration to assess perceived enjoyment or pleasure	Not recommended for EMA but see hedonic motivation (below)
Perceived capability	Perceptions of ability, capacity, or competence to perform a behavior independent of motivation	Not intended to be dynamic	Capabilities in the execution of a physical activity task should not change abruptly in many populations, but clinical populations may experience more dynamic shifts	Not generally recommended for EMA
Perceived opportunity	Perceptions of the physical and social environment that affect access to behavioral engagement independent of motivation	Not originally intended to be dynamic but see window of opportunity (below)	Forecasted perceptions of opportunity are meant to be prospective and manifest of repeated behavior	Not recommended for EMA but see window of opportunity (below)
Intention	Decision to perform a behavior	To understand the short-term (i.e., daily) decisions	Depends on the research question. M-PAC was constructed with intention representing an overall decision about a pattern of behavior. Temporality could be explored if short-term behavior is central to the research question	Not generally recommended for EMA
**Regulatory process**				
Proactive regulation	Anticipated challenges or opportunities, pre-emptive actions to manage thoughts, emotions, and behaviors	Not intended to be dynamic	M-PAC was constructed with proactive regulations as deliberative and prospective; however, these could be explored as dynamic depending on research focus. The temporality of the research question is critical	Not generally recommended for EMA
Reactive regulation	Managing thoughts, emotions, and behaviors in response to unexpected situations, challenges, or changes that have already occurred	Managing thoughts, emotions, and behaviors in response to changing contexts or situations as they arise	Unlike proactive regulation, which involves anticipating and preparing for potential challenges, reactive regulation includes adapting to immediate circumstances after they arise, making it ideal to assess via EMA	Recommended for EMA
Self-monitoring	Ongoing process of observing and evaluating one's thoughts, feelings, behaviors	Can be dynamic or retrospective	M-PAC considers self-monitoring as deploying generalized strategies over time; however, this could be explored as dynamic depending on research focus (e.g., awareness)	Recommend assessment of attentional focus/awareness using EMA
**Reflexive process**				
Habit	Learned cue-behavior associations eliciting an impulse to enact the behavior on exposure to the cue	Determining what is usual or typical behavior during specific times; represented as behavioral frequency by context stability	Construct of habit continues to be difficult to assess; however, EMA and use of GPS data offers an opportunity to triangulate context stability and behavioral frequency with traditional assessments of habit as a psychological factor	Recommended for EMA
Identity	Self-reflection and the perception of one's self in social and personal contexts guiding behavior	Feeling of being yourself reflected through affect or self-congruence	Emotion could reflect the exact moment of identity congruent or discrepant cues, but the longstanding feeling should be a mood of coherence-confusion	Recommended for EMA
“Ongoing affective judgement” reformulated as hedonic motivation	Cue-driven want to perform a behavior (from how pleasant or unpleasant a behavioral outcome is expected to be)	State-based hedonic or affectively-charged motivation with comparator/reinforcing value	Static “ongoing reflective process” was a proxy for hedonic motivation and reinforced value	Recommended for EMA
“Ongoing perceived opportunity” reformulated as window of opportunity	Context-based appraisal of the opportunity to perform a behavior	Near real-time perceptions of accessibility within social and environmental contexts	Static “ongoing reflective process” proxy for actual opportunity	Recommended for EMA

### Reflective processes

Constructs underlying reflective processes are positioned as deliberative, thoughtful processing of information taken from multiple sources of intrapersonal experiences used to arrive at an overall evaluation (Conner and Norman, 2015). While it is conceivable to position these reflective processes as dynamic (e.g., momentary changes in capability, attitude, vacillation in decisional intention), the original conception of such processes in M-PAC was akin to a slower, gradual change in the constructs over time. Therefore, it is recommended that reflective processes be measured in waves commensurate with some distance between time-frames to allow for this slower change, and only to include a dynamic measure if there is reason to expect vast dynamic shifts (e.g., clinical conditions dramatically affecting perceptions of capability; changing contexts dramatically affecting evaluations of a behavior, etc.) or if the temporal-frame itself is a specific feature on the research inquiry (e.g., daily change in attitudes, intentions).

### Regulatory processes

Regulatory processes represent a combination of prospective and reactive tactics (Rhodes and Lithopoulos, 2023). Similar to reflective processes, planning, and overall monitoring are likely more deliberate and prospective in nature, but emotional regulation and attentional focus as a part of the regulatory process are likely to be more dynamic (Karoly, 1992; Carver and Scheier, 1982; Schwarzer, 2008; Duckworth et al., 2016; Mann et al., 2013). In terms of M-PAC, we suggest that planning, overall self-monitoring, and summary emotion regulation may be best assessed in waves commensurate with some distance between time-frames to allow for this slower change in these tactics unless there is reason to expect very dynamic shifts (e.g., a person with a highly variable schedule that necessitates dynamic shifts in day-to-day plans, etc.). By contrast, EMA may be ideal to measure such aspects like the application of acute emotion regulation techniques (Gross, 2014), acceptance and commitment constructs (Hayes et al., 1999), and/or state self-control as *reactive behavioral regulation* (Nigg, 2017; Boemo et al., 2022; Colombo et al., 2020). In addition, we believe the concept of attentional focus/awareness (Kuhl, 1984; Sniehotta et al., 2006), arguably the dynamic component of self-monitoring and action control, should be measured with EMA if possible, because this is likely to better capture awareness than static, infrequent, aggregate assessments.

### Reflexive processes

Assessment of reflexive processes seems conceptually ideal for EMA, yet is challenging because in-the-moment assessment may interrupt the very reflexive nature of the construct under assessment. In other words, self-report requires self-reflective awareness and the willingness to report it—which by definition is no longer reflexive (Sniehotta and Presseau, 2011; Williams and Rhodes, 2023).

Habit converges on three critical elements: behavioral repetition, high degree of automaticity, and cued actions in stable contexts (Orbell and Verplanken, 2015). Wood et al. (2005) consider habit measurement as a multiplicative product of behavior frequency and context stability. Maher et al. (2021) recently investigated the relationships between context stability (assessed by EMA) on self-reported baseline habit and behavioral outcomes assessed through accelerometry and found context stability was associated with PA but not habit. It is possible that a person is performing a behavior with conscious means merely under the same conditions (Rhodes and Rebar, 2018), and this may explain the findings from Maher et al. (2021). However, because habit is a psychological construct, it will also be prudent for researchers to measure habit via self-report (Rebar et al., 2018) through more traditional assessment schedules. Future research integrating frequency-in context measurement approaches to assess momentary habit in combination with traditional habit measures could be useful to triangulate results. This could be captured through technological advances to actively sense (i.e., where participants take a moment to respond to prompts/questions) and passively sense (i.e., wearables that continuously collect information without participants' conscious attention), which holds promise for reflexive constructs like habit.

Assessments of identity through EMA may also pose challenges because it is largely considered a stable construct, resistant to change, and developed over a long period (Burke and Stets, 2022). Assessment of self-categorization as an “active person” multiple times a day seems pointless as it is not likely to change within that day. However, the specific antecedents underlying the identity-behavior relationship (cues, affective states) consistent with the dynamic identity control system (Burke, 2006; Carver and Scheier, 1998) may be better suited for assessment with EMA. For example, behavioral performance that aligns with a person's identity standard is thought to cue positive affect, where behavior that is in misalignment with cues lack of coherence, and have been found to be associated with less positive affect (Strachan et al., 2011; Strachan and Brawley, 2008). Similar to habit, the interaction between behavioral performance multiplied by affective properties could represent the dynamic form of identity. A caveat is that the coordination between affect and behavior could be a mere result of affect regulation (Stevens et al., 2020), whereby PA is engaged in to rectify a less optimal affective state, and not linked to an identity control system. As such, self-congruence may be a better factor within the identity control system to investigate. Self-congruence is defined as the alignment between an individual's behavior, environment, and self-identity or schema (Sirgy et al., 2016), operating as a feedback mechanism, ensuring that behaviors and external cues reinforce one's identity (Yu et al., 2020). Thus, disruptions such as being in novel contexts or having to face conflicting roles can challenge this self-congruency. Together, it is recommended that researchers measure identity via self-report (Rhodes et al., 2016, 2024) using more traditional assessment schedules along with the combination with EMA assessments to best triangulate the identity construct.

#### Repositioning “ongoing reflective constructs” as reflexive constructs for EMA

Interestingly, within the original M-PAC framework, perceived opportunity and affective judgments were positioned as *ongoing reflective processes* because it was posited they could predict intention and the translation of an intention into behavior, to the extent that they represent a proxy for the affective and logistical factors that challenge one's competing behavioral decisions over the course of one's day (Rhodes, 2017). In the consideration of how M-PAC could be best adapted for application of EMA methodologies, we realize that intensive longitudinal sampling represents an ideal way to re-explore and re-interpret this theorizing. In particular, affective judgements map onto recent work focusing on affectively charged or hedonic motivation (Williams and Rhodes, 2023; Williams, 2018), representing this dynamical affective influence on PA at a micro-timescale. Specifically, affectively charged motivation includes aspects of dread or want, compared to other behavioral options in the moment (Williams, 2019; Williams and Evans, 2014; Stults-Kolehmainen et al., 2023). This form of affect toward a behavior is considered dynamic, dependent on situational cues and less cognitively processed than aggregate affective judgments (Stevens et al., 2020).

Similarly, assessments of the window of opportunity to engage in a behavior like PA involve the dynamic context of the situation (Dunton, 2017). Thus an EMA measure of perceived opportunity could demonstrate some important contextual variability when compared to a static, anticipatory, and aggregate perceived opportunity measure (Lithopoulos et al., 2023). Despite the divergence from the original conceptualization of the reflective process within M-PAC, we believe that the dynamic re-operationalization of ongoing reflective constructs are complementary to EMA design. Concurrent nomothetic and idiographic assessments of these factors could yield some important insights. The ability to capture these constructs in context, functioning as a part of the reflexive process (i.e., changes in hedonic motivation, window of opportunity), assessed in concert with the slow, deliberate formation of a behavioral evaluation (affective judgments, perceived opportunity) will enrich the utility of the M-PAC framework.

## Early adaptations of M-PAC for EMA

Although research applying M-PAC within EMA studies is in its infancy, some examples of application have begun to emerge. For example, Kwan et al. (2020) conducted a longitudinal cohort study that involved adolescents completing up to five EMA prompts daily during a seven-day sampling period, assessed annually over 4 years. A one-item state motivation question was used as a proxy of *hedonic motivation*, one self-control item was selected as a *reactive behavioral regulation* measure, and a proxy *habi*t measure included the degree to which current behaviors aligned with typically behavioral patterns during that time of day. Investigations using these EMA data explored how these M-PAC-based variables predicted PA during the after-school period (Kwan et al., 2022). Results of the study found *hedonic motivation* was a consistent significant predictor of acute PA, while *reactive behavioral regulation* was a significant predictor of acute PA only during the immediate after-school period (3:30 PM−6:00 PM). Conversely, proxy *habit* was a significant predictor of PA only during the late evening period (8:30 PM−10:00 PM). Overall, findings underscore the importance of temporality, suggesting that different M-PAC constructs may exert varying levels of influence on PA at different times of day.

Utilizing this same data-set, Harris et al. (2024) examined the impact of within- and between-subject effects of the M-PAC based assessments on acute PA (defined as the 60 min following each prompt assess by accelerometers). Findings revealed that overall higher levels of *hedonic motivation*, and times where adolescents were experiencing higher levels of *hedonic motivation* when compared to one's typical levels, were associated greater acute PA behaviors. Engagement with activities less consistent with usual *habit* were also found to be associated with PA. Overall, results highlight that momentary motivational processing and deviation from average habitual processing can drive increased PA, while *reactive regulatory* processing, often associated with resisting temptations, may have a lesser impact on acute PA.

Finally, a study by King-Dowling et al. (2025) applied M-PAC-based EMA questions to assess *hedonic motivation* and *habit* among adolescents and young adult (AYA) cancer survivors. Within-subject *hedonic motivation* had a significant effect on acute PA, reinforcing the idea that fluctuations in hedonic motivations, considering other factors such as fatigue and pain, play a critical role in predicting when and how much these AYA participate in PA. While these studies provide initial evidence for the utility of M-PAC in dynamic modeling of PA, there are caveats with respect to the reliability and validity of the measures included, and a lack of integration of both traditional and EMA assessments examined in concert, reinforcing the need for this current paper.

## Future directions and unresolved issues

Technological advancements have outpaced theoretical development in behavioral sciences, giving rise to real-time data collection methods like EMA to better understand PA. Thus, it is imperative to revisit and incorporate theories that contextualize these findings and provide a structured approach for designing interventions. The M-PAC framework (Rhodes, 2017, 2021) holds significant conceptual promise for EMA research, with this paper outlining how EMA measures of reflective, regulatory, and reflexive processes may be re-positioned and integrated with traditional forms of assessment. Still, there are several unresolved issues and thus considerable future directions needed for research.

A priority issue for consideration in adapting any behavioral theory for EMA is what measures to include in EMA assessment and what measures to retain in traditional assessment schedules. In this review, we outlined that *reflective processes*, positioned as deliberative, thoughtful processing of information taken from multiple sources of intrapersonal experiences, are not applicable to EMA unless specific circumstances inform the research question otherwise. *Regulatory processes*, by contrast, have utility in EMA assessment, when positioned as reactive behavioral regulation (e.g., acute emotion regulation), or attentional focus/awareness on enacting the intended behavior, *and reflexive processes* (i.e., cue-triggered responses) are conceptually ideal for EMA. In summarizing the early application of M-PAC in EMA studies, the extant research provides initial proof of concept evidence for the utility of M-PAC in dynamic modeling of PA. However, future research is certainly needed to assess the predictive and complementary scope of proposed M-PAC constructs, and to extend this beyond adolescents and to apply with broader demographics and populations.

Related to selection of measures relevant to EMA methodologies, is the critical challenge of how to best operationalize constructs using intensive longitudinal assessments. Along these lines, we believe the application of EMA also represents an ideal way to re-explore and re-interpret M-PAC constructs of affective judgments and perceived opportunity, originally positioned as *ongoing reflective processes* but re-operationalized to reflexive process measures as context-based representations of hedonic motivation and window of opportunity. Future research is now needed to explore and test the unique predictive efficacy of such a re-interpretation, including within the larger M-PAC framework of constructs. Reconsiderations in how other traditional behavioral theory constructs are operationalized for EMA are also recommended (Conroy et al., 2020).

Integration of both trait-based M-PAC measures and dynamic measures has been recommended in this review (see Figure 1 as example design), yet the ongoing validation of EMA measures following established standards (see Messick, 1995) and subsequent analyses methods to model the blend of nomothetic (generalized) and idiographic (individualized) approaches is warranted in sustained future research (see Ruissen et al., 2021). It is prudent that we re-think the application of traditional behavior change theories to apply new multivariate, Bayesian, control systems modeling, or machine learning approaches to predicting behavioral outcomes such as PA across timescales. These approaches will help strengthen the M-PAC framework's ability to explore the dynamic and complex interplay of psychological processes determining PA behavior across different timescales.

Finally, the opportunities to combine active sensing through EMA with passive data collection from wearable technologies also offers potential for significant discoveries. As indicated in Figure 1, this integration may better capture the potential interactions between psychological processes and real-time behavior, along with biofeedback, GPS, and environmental information to further advance the field of PA research. This is an exciting time in behavioral sciences, and technological advances are providing us new tools capable of collecting copious amounts of data to be explored. We recommend the use of this paper as a “version 1.0” starting point, acknowledging the continuing need to assess its validity and reliability properties and invite authors to revise as more evidence is available.
