# Supplementary material for: Dynamic representations of theory testing in physical activity using ecological momentary assessment: an example guide utilizing multi-process action control
Source: Front Psychol. 2025 Mar 28;16:1547090. doi: 10.3389/fpsyg.2025.1547090 (PMC11985800; doi:10.3389/fpsyg.2025.1547090)
Supplement: Supplementary file 1 [file Table_1.docx]

**Supplementary Table:**

This initial user guide serves as a foundation and provides recommendations for adapting the M-PAC framework into a practical and feasible measure for EMA studies. Below is a list of suggested measures that align with these recommendations; however, it should be noted that this is not an exhaustive review of all possible measures.

| M-PAC Construct | Existing Measure | Potential adapted EMA questions |
| --- | --- | --- |
| *Regulatory Process* | |  |
| Reactive Regulation | Adapted from Brief State Self-Control Scale (Tangney et al,, 2004) | ***Example question:***   - *If I were tempted by something other than PA right now, it would be very difficult to resist*   7-point scale ranging from 1 (*Not true*) to 7 (*Very true*) |
|  | State Self-Control Capacity Scale (Bertrams, et al., 2011) | ***Example question: “Currently…”***   - *I have to force myself to stay focused on my PA plans* - *I'm having trouble pulling myself together to do PA*   7-point scale from 0 (*not at all*) to 6 (*very much so*) |
|  | Adapting Coping Flexibility Scale (CFS; Kato, 2012) | ***Example question: “At this time…”***   - *If I feel that I have failed to cope with stress, I change the way in which I deal with stress to fulfil my PA plans* - *When a stressful situation has not improved, I try to think of other ways to cope, so I can do PA*   4-point scale from 1 (*not applicable*) to 4 (*very applicable*) |
| *Reflexive Processes* | |  |
| Hedonic (affectively-charged) motivation | Motivation of acute physical activity Crosley-Lyons et al., (2023) | ***Example Question:***   - *How do you feel about engaging in PA in the next 15 min?*   VAS ranging from 0 (*dreading it*) to 100 (*excited to do it*) |
|  | CRAVE scale (Stults-Kolehmainen et al. 2021; Filgueiras et al., 2023) | ***Example Question:***   - *Currently, I have a desire to be physically active*   11-point Likert Scale ranging from 0 (*not at all*) to 10 (*more than ever*) |
| Window of Opportunity | Question of perceived opportunity (see Aartolahti et al., 2021) | ***Example Question:***   - *Do you have opportunities for PA right now?*   5-point scale from 1(*not possible*) to 5 (*very possible*) |
|  | Question of perceived control (see Lithopoulos et al., 2023) | ***Example questions:***   - *At this moment, I believe I have the opportunity to engage in PA over the next few hours* - *At this moment, I could find a way to fit PA in my schedule so that I can be active*   7-point scale from 1 (*strongly disagree*) to 7 (*strongly agree*) |
|  | Adapted from question of self-control in alcohol use (see Remmerswaal, 2019) | ***Example question:***   - *How much control do you feel you have over your ability to be active*   100-point scale from 0 (*no control*) to 100 = *a lot of control*) |
| Habit | Context stability questions (see Stojanovic et al., 2022) | ***Example question:***   - *Physical place facet: I did my last PA in the same physical surroundings in which I did my earlier PA as well* - *Time facet: I did my last PA at the same time in the course of the day at which I usually do PA* - *General context: The context (physical as well as mental) of my last bout of PA was exactly the same as earlier PA*   11-point scale from 0 (*does not apply at all*) to 10 (*applies perfectly*) |
|  | Measures of physical, social, and environmental contexts (see Dunton et al., 2007) | *Example question:*   - What are you currently doing? - Where are you currently? - Who are you currently with?   Creation of response items |
| Identity | Self-Congruence (see Sirgy et al. 1997)  Positive/Negative Affect Scores (see Do et al., 2021) | ***Example question:***   - Take a moment to think about your current feelings about yourself. Indicate your agreement or disagreement to the following statement: I’m currently feeling and behaving in a manner that is consistent with how I see myself?”   0 = completely disagree to 10 = completely agree   - How are you currently feeling in relation to your true self?   0 = not at all like my true self to 10 = completely like my true self  ***Example questions:***  Right now, how (*affect term*) do you feel?   - Positive-activated affect (*happy*, *energetic*), positive-deactivated affect (*relaxed*), negative-activated affect (*tense*, *stressed*), and negative-deactivated affect (*sad*, *fatigued*)   11-point scale from 0 (*does not apply at all*) to 10 (*applies perfectly*) |
|  | PANAS-C (Ebesutani et al., 2012; Laurent et al., 1999) also see Dunton et al., 2019  Acute mood state (see van Rijsbergen et al., 2012) | ***Example question:***   - Positive affect: How (HAPPY, JOYFUL) were you feeling just before the beep went off? - Negative affect: How (STRESSED, MAD ORANGRY, NERVOUS OR ANXIOUS, SAD) were you feeling just before the beep wet off?   4-point scale from 0 (*not at all*) to 3 (*extremely*)  ***Example question:***   - *Please rate your current mood on a scale of 1 to 10*   10-point scale from 1 (*sad*) to 10 (*happy*) |

**Notes:** PA = Physical activity. One of the key challenges in EMA research lies in balancing the depth and breadth of information sought with the constraints imposed by the limited number of items that can reasonably be included, especially when participants are required to respond to multiple prompts each day. The use of single items derived from multi-item measures with established validity and reliability offers a potential solution, albeit with limitations. Research by Song and colleagues (2023), for example, provides evidence that some single-item measures can exhibit moderate to strong correlations with their corresponding multi-item scales, indicating good concurrent validity. Furthermore, while single-item measures have demonstrated significant predictive validity, the strength of these predictions is generally lower compared to their multi-item counterparts. This underscores the importance of careful consideration when selecting and adapting measures for EMA studies to ensure both feasibility and data quality. An additional consideration is around the response options for EMA questions, and it is suggested that adaptations be made in order to maintain consistency between all questions being asked. VAS scales that have been used previously (see Do et al., 2021) should be considered given that it can capture maximal variability in responses being captured multiple times each day.

**References**

Aartolahti, E., Eronen, J., Törmäkangas, T., Rantanen, T., Hirvensalo, M., Palmberg, L., ... & Rantakokko, M. (2021). Perceived opportunities for physical activity and willingness to be more active in older adults with different physical activity levels. *International journal of environmental research and public health*, *18*(11), 6146.

Bertrams, A., Unger, A., & Dickhäuser, O. State Self-Control Capacity Scale--German Version. *Zeitschrift für Pädagogische Psychologie/German Journal of Educational Psychology*.

Crosley-Lyons, R., Do, B., Hewus, M., & Dunton, G. F. (2023). An ecological momentary assessment study of affectively-charged motivational states and physical activity. *Psychology of Sport and Exercise*, *67*, 102423.

Do, B., Rhodes, R. E., Kanning, M., Hewus, M., & Dunton, G. F. (2022). Examining whether affectively-charged motivations predict subsequent affective response during physical activity: an ecological momentary assessment study. *Frontiers in Sports and Active Living*, *4*, 1029144.

Dunton, G. F., Whalen, C. K., Jamner, L. D., & Floro, J. N. (2007). Mapping the social and physical contexts of physical activity across adolescence using ecological momentary assessment. *Annals of Behavioral Medicine*, *34*(2), 144-153.

Filgueiras, A., Stults-Kolehmainen, M. A., Boullosa, D., Sinha, R., Bartholomew, J. B., McKee, P., ... & Ash, G. I. (2023). The CRAVE and ARGE scales for motivation states for physical activity and sedentarism: Brazilian Portuguese translation and single-item versions. *Frontiers in Psychology*, *14*, 1106571.

Harris, S., Brown, D., King-Dowling, S., Cairney, J., & Kwan, M. (2024). Examining real-time physical activity in adolescents using the Multi-Process Action Control Model: An ecological momentary assessment study. *Current Issues in Sport Science (CISS)*, *9*(1), 005-005.

Kato, T. (2012). Development of the Coping Flexibility Scale: evidence for the coping flexibility hypothesis. Journal of counseling psychology, 59(2), 262.

Lithopoulos, A., Zhang, C. Q., Williams, D. M., & Rhodes, R. E. (2023). Development and validation of a two-component perceived control measure. *Annals of Behavioral Medicine*, *57*(2), 175-184.

Remmerswaal, D., Jongerling, J., Jansen, P. J., Eielts, C., & Franken, I. H. (2019). Impaired subjective self-control in alcohol use: An ecological momentary assessment study. *Drug and alcohol dependence*, *204*, 107479.

Stojanovic, M., Grund, A., & Fries, S. (2022). Context stability in habit building increases automaticity and goal attainment. *Frontiers in Psychology*, *13*, 883795.

Schöndube, A., Bertrams, A., Sudeck, G., & Fuchs, R. (2017). Self-control strength and physical exercise: An ecological momentary assessment study. *Psychology of Sport and Exercise*, *29*, 19-26.

Sirgy, M. J., Grewal, D., Mangleburg, T. F., Park, J. O., Chon, K. S., Claiborne, C. B., ... & Berkman, H. (1997). Assessing the predictive validity of two methods of measuring self-image congruence. *Journal of the academy of marketing science*, *25*, 229-241.

Song, J., Howe, E., Oltmanns, J. R., & Fisher, A. J. (2023). Examining the concurrent and predictive validity of single items in ecological momentary assessments. *Assessment*, *30*(5), 1662-1671.

Stults-Kolehmainen, M. A., Blacutt, M., Fogelman, N., Gilson, T. A., Stanforth, P. R., Divin, A. L., ... & Sinha, R. (2021). Measurement of motivation states for physical activity and sedentary behavior: development and validation of the CRAVE scale. *Frontiers in Psychology*, *12*, 568286.

Tangney, J. P., Baumeister, R. F., & Boone, A. L. (2004). High self-control predicts good adjustment, less pathology, better grades, and interpersonal success. *Journal of Personality*, 72, 271–324.
